# Treating Supraventricular Tachycardia With Amiodarone in a Patient With Ebstein’s Anomaly

**DOI:** 10.7759/cureus.33772

**Published:** 2023-01-14

**Authors:** Mohammed Y Alnami, Hassan M Hassan Almalki, Nadher M Hassan Hakami, Omar E Masmali

**Affiliations:** 1 Pediatric Emergency Medicine, King Fahad Central Hospital, Jazan, SAU; 2 Pediatric Emergency Medicine, Jazan General Hospital, Jazan, SAU

**Keywords:** ebstein's anomaly, arrhythmia, amiodarone, supraventricular tachycardia, ebstein, anomaly

## Abstract

Ebstein's anomaly is a congenital deformity marked by disease of the tricuspid valve and right cardiac hypertrophy. The severity, morphology, and appearance of Ebstein's anomaly cases might vary greatly. We discuss a case of an eight-year-old child with Ebstein's anomaly who presented with supraventricular tachycardia and was effectively treated with amiodarone after initial treatment with adenosine failed to reduce the heart rate.

## Introduction

In 1866, Wilhelm Ebstein discovered Ebstein's anomaly (EA) while studying the heart of a 19-year-old laborer who had died from tricuspid regurgitation caused by a severe malformation of the tricuspid valve [1]. EA is characterized by the abnormal positioning of the posterior and septal leaflets of the tricuspid valve. These leaflets are displaced in a spiral manner toward the base of the right ventricle, a phenomenon that is frequently accompanied by the presence of an atrial septal defect or other cardiac malformation [2].

EA is characterized by various pathological abnormalities in the heart, including an enlarged right atrium and right ventricle, as well as the presence of atrioventricular (AV) accessory conduction pathways (APs). These abnormalities create a favorable environment for the emergence of supraventricular and ventricular tachyarrhythmias, which refer to abnormal heart rhythms occurring at unusually high rates [3].

Supraventricular tachycardia (SVT) can be terminated through the use of vagal maneuvers, such as the Valsalva maneuver or carotid sinus massage, or through the administration of adenosine or cardioversion [3]. However, in some cases, tachycardia may recur, necessitating further management options.

In this case, we successfully treated SVT in a child with EA with amiodarone, a class III antiarrhythmic drug, after the child failed to respond to first-line adenosine in an emergency setting.

## Case presentation

**Figure 1:**
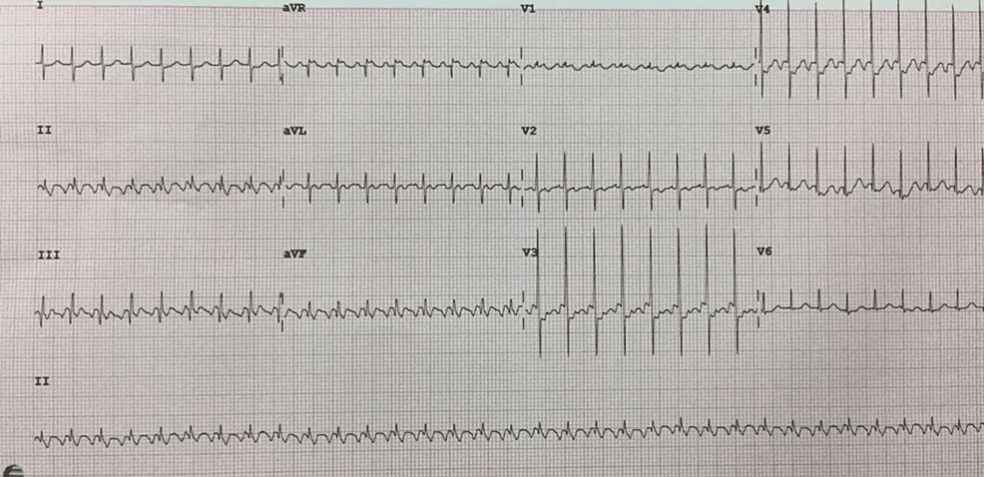
Electrocardiogram showing supraventricular tachycardia in the patient on first admission

An eight-year-old boy known to have EA with severe tricuspid regurgitation, who was receiving regular diuretics, presented to the emergency department with a history of fever, vomiting, and palpitation for the past three days. On examination, he was an unwell child with central cyanosis, a heart rate of 240 bpm, oxygen saturation of 100%, and blood pressure of 107/84 mmHg. His cardiovascular examination revealed a pansystolic murmur in the tricuspid area. An ECG was performed and showed SVT (Figure 1).

**Figure 2:**
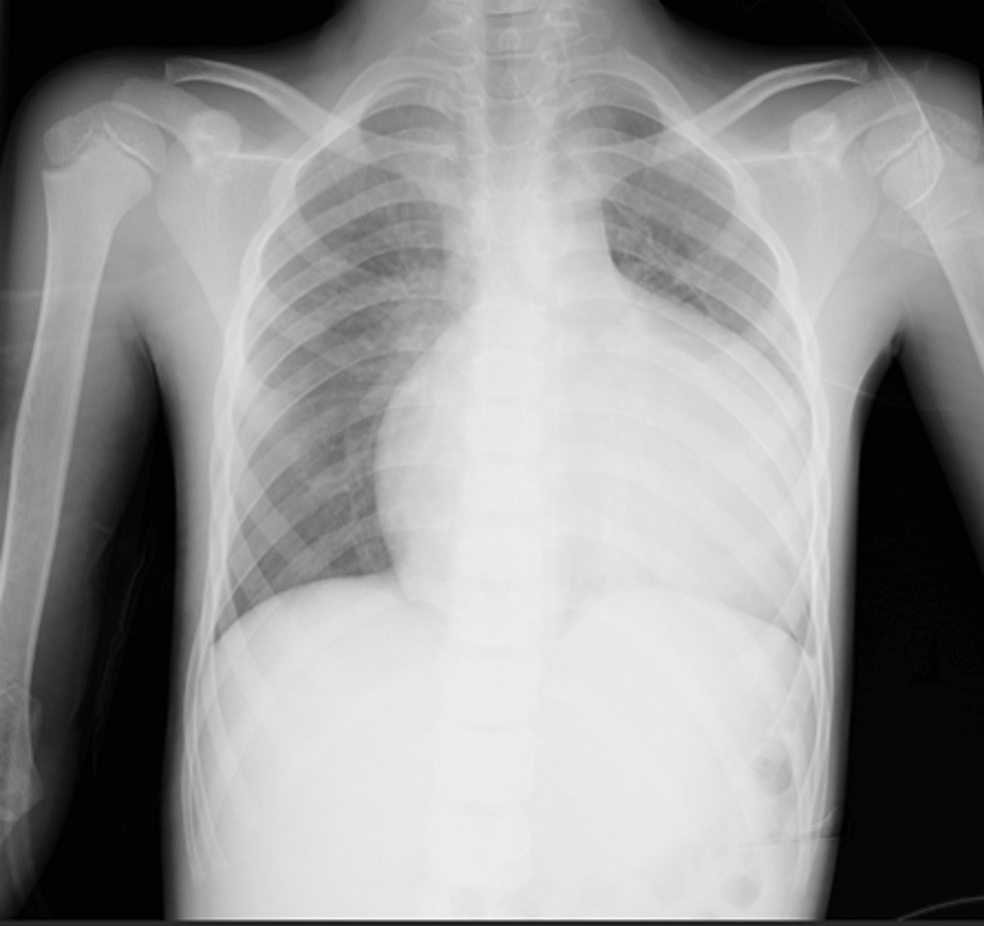
Chest X-ray showing massive cardiomegaly and hilar congestion

Two doses of adenosine (first dose: 0.1 mg/kg; second dose: 0.2 mg/kg) were given, and he reverted to a normal rate. An X-ray showed massive cardiomegaly and hilar congestion (Figure 2).

Laboratory investigations of the patient are shown in Table 1. The patient was kept nil per mouth and started on intravenous fluid, antibiotics (cefotaxime 50 mg/kg/day and azithromycin 10 mg/kg/day), and beta-blockers (Inderal 15 mg). He remained vitally stable. An echocardiogram was repeated and showed severe EA with severe tricuspid regurgitation and dilated right atrium and right ventricle. The patient was sent home on Lasix 1 mg/kg/dose twice daily and Inderal 2 mg/kg/day three times daily, with instruction to return for a follow-up in three months.

**Table 1:** Laboratory investigations of the patient on first and second admission BUN: blood urea nitrogen; COVID-19: coronavirus disease 2019; PCR: polymerase chain reaction.

Investigations	1^st^ admission			2^nd^ admission				
	Day 1	Day 2	Day 3	Day 1	Day 2	Day 3	Day 4	Day 5
Hemoglobin (mg/dL)	12.8	12.9	12.4	14	13	14.2	13.6	14.3
WBC (x10^9^/L)	5.38	4.29	6.2	6.14	6.42	4.39	6.94	7.48
Platelets (x10^9^/L)	362	204	335	297	245	231	353	349
Sodium (mEq/L)	138	-	-	137	-	-	-	-
Potassium (mEq/L)	4.8	-	-	3.6	-	-	-	-
BUN (mg/dL)	4.7	-	-	1.4	-	-	-	-
Creatinine (mg/dL)	57	-	-	50	-	-	-	-
Troponin I (ng/ml)	-	7	-	-	1.1	-	-	-
Albumin (g/dL)	-	-	-	-	-	39	-	-
Phosphorous (mg/dL)	-	-	-	-	-	1.9	-	-
Magnesium (mEq/L)	-	-	-	-	-	0.8	-	-
Alanine aminotransferase (U/L)	-	11	-	-	15	-	-	-
Aspartate transferase (U/L)	-	33	-	-	28	-	-	-
COVID-19 PCR	Negative	-	-	-	-	-	-	-

**Figure 3:**
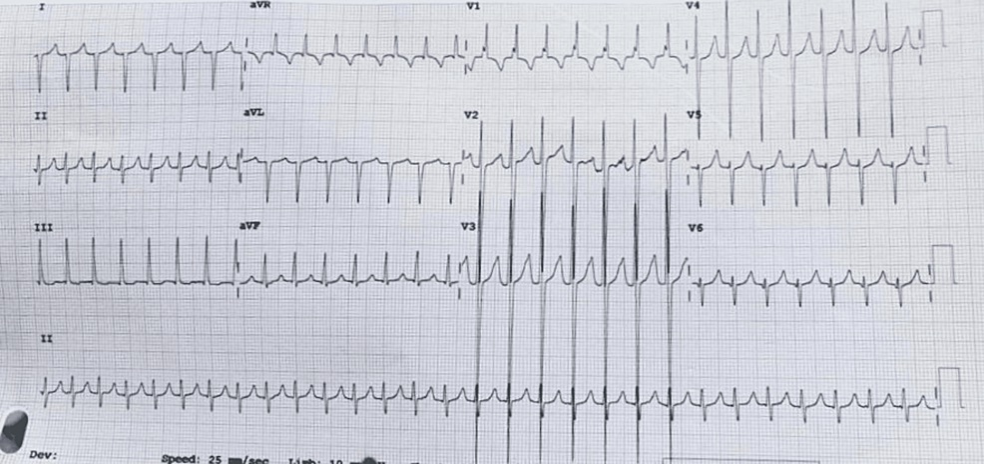
Electrocardiogram showing supraventricular tachycardia in the patient on second admission

The patient missed the follow-up appointment and came to the emergency department after months with one day of palpitation and vomiting. The examination showed mild dehydration, a heart rate of 217 beats per minute, and a widely split first heart sound with a systolic murmur. An ECG was performed and showed SVT (Figure 3).

Four doses of adenosine were given, but there was no change in the heart rate. We planned for direct current (DC) cardioversion, but the parents did not give consent, so one dose of amiodarone 5 mg/kg was administered. The patient’s heart rate improved from 217 to 160. He was started on an amiodarone infusion and transferred to the pediatric intensive care unit (PICU). During his stay, his heart rate fluctuated and increased to 190 beats per minute, so the amiodarone dose was increased to 6 mg/kg/minute. He stayed in the PICU for 24 hours during which his heart rate returned to normal, and the infusion was stopped. His laboratory investigations on the second admission are shown in Table 1. He was transferred to the ward and started on metoprolol 25 mg twice daily and Lasix 1 mg/kg/dose twice a day. The patient remained stable during treatment and was eventually discharged from the hospital with a follow-up plan. Upon following up, it was determined that the patient had remained well and had not experienced any further episodes of SVT in the last three months.

Initial submission of this case to Cureus occurred on January 2, 2023.

## Discussion

The incidence of EA occurs in one to five of 200,000 live births and constitutes less than 1% of congenital disorders. Patients with EA may present with a range of symptoms, which can depend on various hemodynamic factors such as the severity of tricuspid regurgitation, the degree of right-to-left shunting through a patent foramen ovale or atrial septal defect, and the degree of right ventricular dysfunction. Furthermore, EA is often accompanied by SVT and can be particularly dangerous in individuals with EA due to the associated hemodynamic and anatomic abnormalities [4].

The management of SVT in EA can be challenging due to the complexity of the underlying cardiac anatomy and the potential for concomitant heart failure and other cardiac complications [5]. SVT in EA can be caused by accessory pathways, AV conduction delay, and atrial tachyarrhythmias. The management of SVT in EA requires a multidisciplinary approach that may include pharmacological treatment, catheter ablation, and surgical intervention [6].

Surgical or catheter-based interventions may be recommended for patients with symptoms, although these procedures can be challenging. However, they remain highly effective and curative options for management. Without intervention, these patients are at higher risk of sudden cardiac death rather than heart failure, so pharmacological prevention of SVT may be a reasonable approach [7].

Adenosine is the first line in the management of SVT, particularly atrioventricular nodal reentrant tachycardia (AVNRT) and atrioventricular reentrant tachycardia (AVRT). However, adenosine may not be effective in all patients with SVT in EA [8]. Amiodarone is a class III antiarrhythmic medication that works by inhibiting the conduction of abnormal electrical signals through the heart [9]. There have been numerous reports documenting the effectiveness of amiodarone in treating SVT. In a comparative study by Hill et al. [10], amiodarone was used to treat various tachyarrhythmias in children, resulting in a success rate of 80%. Another study also demonstrated the efficacy of amiodarone in treating SVT in patients with congenital anomalies [11]. The literature is scarce regarding the use of amiodarone in the treatment of SVT in EA. However, one case of treating atrial fibrillation (AF) with amiodarone in EA has been reported [12]. Moreover, this is the first case of the use of amiodarone in the management of SVT in EA in Saudi Arabia.

## Conclusions

In a patient with SVT, the recommended therapy is the administration of IV adenosine, cardioversion, and catheter ablation. Our case showed that amiodarone is extremely effective in the treatment of children with SVT that is unresponsive to conventional antiarrhythmic drugs. The drug does not worsen the existing condition and is, therefore, excellent for the treatment of patients with EA.

## Appendices

Peer review for this article began on 1/02/2023.
